# Discovery of a microbial transglutaminase enabling highly site-specific labeling of proteins

**DOI:** 10.1074/jbc.M117.797811

**Published:** 2017-07-27

**Authors:** Wojtek Steffen, Fu Chong Ko, Jigar Patel, Victor Lyamichev, Thomas J. Albert, Jörg Benz, Markus G. Rudolph, Frank Bergmann, Thomas Streidl, Peter Kratzsch, Mara Boenitz-Dulat, Tobias Oelschlaegel, Michael Schraeml

**Affiliations:** From ‡ Roche Diagnostics GmbH, CPS, Nonnenwald 2, 82377 Penzberg, Germany,; § Roche Sequencing, NimbleGen, Madison, Wisconsin 53719, and; ¶ F. Hoffmann-La Roche Ltd., pRED, Grenzacherstrasse 124, 4070 Basel, Switzerland

**Keywords:** antibody engineering, biotechnology, enzyme structure, high-throughput screening (HTS), peptide array, transglutaminase, Kutzneria albida, antibody-drug conjugates, bio-orthogonal, site-specific conjugation

## Abstract

Microbial transglutaminases (MTGs) catalyze the formation of Gln–Lys isopeptide bonds and are widely used for the cross-linking of proteins and peptides in food and biotechnological applications (*e.g.* to improve the texture of protein-rich foods or in generating antibody-drug conjugates). Currently used MTGs have low substrate specificity, impeding their biotechnological use as enzymes that do not cross-react with nontarget substrates (*i.e.* as bio-orthogonal labeling systems). Here, we report the discovery of an MTG from *Kutzneria albida* (KalbTG), which exhibited no cross-reactivity with known MTG substrates or commonly used target proteins, such as antibodies. KalbTG was produced in *Escherichia coli* as soluble and active enzyme in the presence of its natural inhibitor ammonium to prevent potentially toxic cross-linking activity. The crystal structure of KalbTG revealed a conserved core similar to other MTGs but very short surface loops, making it the smallest MTG characterized to date. Ultra-dense peptide array technology involving a pool of 1.4 million unique peptides identified specific recognition motifs for KalbTG in these peptides. We determined that the motifs YRYRQ and RYESK are the best Gln and Lys substrates of KalbTG, respectively. By first reacting a bifunctionalized peptide with the more specific KalbTG and in a second step with the less specific MTG from *Streptomyces mobaraensis*, a successful bio-orthogonal labeling system was demonstrated. Fusing the KalbTG recognition motif to an antibody allowed for site-specific and ratio-controlled labeling using low label excess. Its site specificity, favorable kinetics, ease of use, and cost-effective production render KalbTG an attractive tool for a broad range of applications, including production of therapeutic antibody-drug conjugates.

## Introduction

Conventional chemical strategies for the modification of therapeutic and diagnostic proteins often lack site specificity, linkage stability, and stoichiometric control, giving rise to heterogeneous conjugates that may cause interference (*e.g.* with immunoreactivity or with the stability of a therapeutic agent).

The industrial development of therapeutic and diagnostic reagents in the coming years will see a massive increase in sophisticated applications requiring stable and truly site-specific conjugation. Research into novel enzymatic methods offering an attractive and cost-effective alternative to established chemical strategies is therefore of paramount importance (1–3).

Microbial transglutaminase (MTG),^2^ first described by researchers of Ajinomoto Co., Inc. in 1989 (4) is one of the most widely used enzymes for the cross-linking of proteins and peptides in many food and biotechnological applications (5–7). MTG was first discovered in and later extracted from the organism *Streptomyces mobaraensis*, and recombinant *S. mobaraensis* MTG represents the bulk of industrially used MTGs today (6).

Transglutaminases catalyze the formation of a stable isopeptide bond between an acyl group (*e.g.* a glutamine side chain) and an alkyl amine (*e.g.* a lysine side chain). In the absence of reactive amine groups, the enzymatic reaction with water leads to deamination of glutamine side chains (5, 8, 9). In contrast to mammalian transglutaminases, bacterial enzymes do not require cofactors, such as Ca^2+^ or GTP, and function over a broad range of pH, buffers, and temperatures (7).

Most characterized MTGs are from *Streptomyces* or *Bacilli*. They share high sequence homology and have similar substrate specificities with typical molecular masses of ≥38 kDa. Being a cross-linking enzyme involved in spore coat formation, MTG displays broad substrate specificity for both acyl donor and alkyl amine groups. Although approaches for the high-throughput screening of improved transglutaminase substrates via phage panning or mRNA display have been reported (10, 11), no comprehensive peptide array-based approaches of peptides larger than trimers (12) have been conducted. Because only the substrate specificities of the enzyme from *S. mobaraensis* and homologous enzymes are known, a bio-orthogonal conjugation approach (*e.g.* simultaneous labeling of a biomolecule using two or more different label substrates and two or more transglutaminase species) is currently not possible.

This work describes the biochemistry, substrate identification, and crystal structure of an active, highly compact (26-kDa) MTG from *Kutzneria albida (*KalbTG) unrelated to any other MTG. The recombinant production of KalbTG in *Escherichia coli* in the presence of its natural inhibitor NH_4_^+^ enabled the high-throughput screening of substrate peptides via peptide array (13). Specificity of this novel transglutaminase for the array-determined substrate sequences is demonstrated by efficient incorporation of labels into engineered target molecules and the poor or undetectable turnover of the enzyme with substrates recognized by conventional MTGs. The high activity and low molecular mass of KalbTG signifies a key advantage for mass production and enzymatic labeling purposes.

Together, these properties make KalbTG highly attractive for a broad range of applications, including the versatile, cost-effective, and site-specific conjugation of biomolecules with various label molecules (*e.g.* production of therapeutic antibody-drug conjugates or chemiluminescent antibodies for *in vitro* diagnostic purposes).

## Results

### Discovery of a novel microbial transglutaminase

Using the amino acid sequence of *S. mobaraensis* protein–glutamine γ-glutamyltransferase as a query, a search for homologs of this enzyme was performed. This yielded the hypothetical gene product KALB_7456 from bacteria *K. albida* DSM 43870, a spore-forming Gram-positive bacterium that was sequenced in 2014 (14). Comparing the primary structures of the *S. mobaraensis* and *K. albida* gene products showed 30% similarity with a distinct conservation of active site residues (supplemental Fig. 1*A*), indicating that the structure and function of this enzyme may be preserved. The *K. albida* gene product is significantly smaller than MTG, amounting to a calculated molecular mass of 30.1 kDa and a molecular mass of 26.4 kDa in the active form. Because MTG is produced as inactive proenzyme and processed by extracellular proteases, such as dispase, to yield the 38-kDa active form (15, 16), we assumed a similar activation mechanism for the hypothetical *K. albida* microbial transglutaminase and used the ProP 1.0 server to analyze the probability for signal and propeptide sequences in the N-terminal region of the protein (supplemental Fig. 1*B*). VAAPTPR↓AP was the only predicted propeptide cleavage site, which corresponds fairly well with the dispase site SAGPSFR↓AP in MTG but putatively has no dispase reactivity because Phe is a required residue in the dispase recognition motif (17, 18). Additionally, a signal peptide is predicted by the ProP 1.0 server with a high-probability cleavage site, GLPTLIA↓TT. However, this sequence bears no resemblance to the significantly longer MTG presequence or other known signal peptides.

### Parallel construct evaluation allows economical recombinant production of KalbTG

To rapidly screen expression conditions for the hypothetical transglutaminase KalbTG, we inserted the synthetic gene into multiple expression vectors designed for the soluble cytosolic or periplasmic expression in *E. coli* using the fragment exchange system (19). Initial screening in the 5-ml scale provided clear evidence that proteins with the anticipated electrophoretic mobility of full-length KalbTG fusions were expressed and that a fusion with tandem SlyD chaperones as described previously (20) yielded the highest amount of soluble protein among all of the constructs tested (data not shown). By suppressing the toxic cross-linking activity of elevated KalbTG concentrations with the addition of ammonium salts, the production process was adaptable to a 10-liter fermenter scale. Purification by standard methods (Q-Sepharose, nickel-nitrilotriacetic acid) yielded several hundred mg of a highly pure enzyme preparation. The purified and activated enzyme remained stable at 4 °C and over multiple freeze–thaw cycles, with the melting point determined at 48.9 °C using dynamic scanning calorimetry (DSC) (supplemental Fig. 2).

### The NimbleGen peptide array delivers a holistic and reproducible approach for transglutaminase substrate discovery

We confirmed that both the SlyD-fused and the mature KalbTG possess basic microbial transglutaminase activity of at least 1.65 units/mg by assaying it with the Zediras MTG-ANiTA kit (M001, compared with 4.30 units/mg by the MTG supplied with the kit and a 0.07-unit/mg blank value with BSA). Next, we searched for specific recognition motifs by assaying KalbTG with the NimbleGen peptide array technology. The turnover of the transamidation reaction between 1.4 million unique 5-mer peptides and biotinylated amine donor *N*-(biotinyl)cadaverine used as a substitute for a Lys substrate was quantified via fluorescence measurement of CyTM5–streptavidin binding.

A control experiment with only CyTM5–streptavidin did not show any significant binding due to the fact that all potential streptavidin binders were removed from the 5-mer array design.

**Figure 1.**
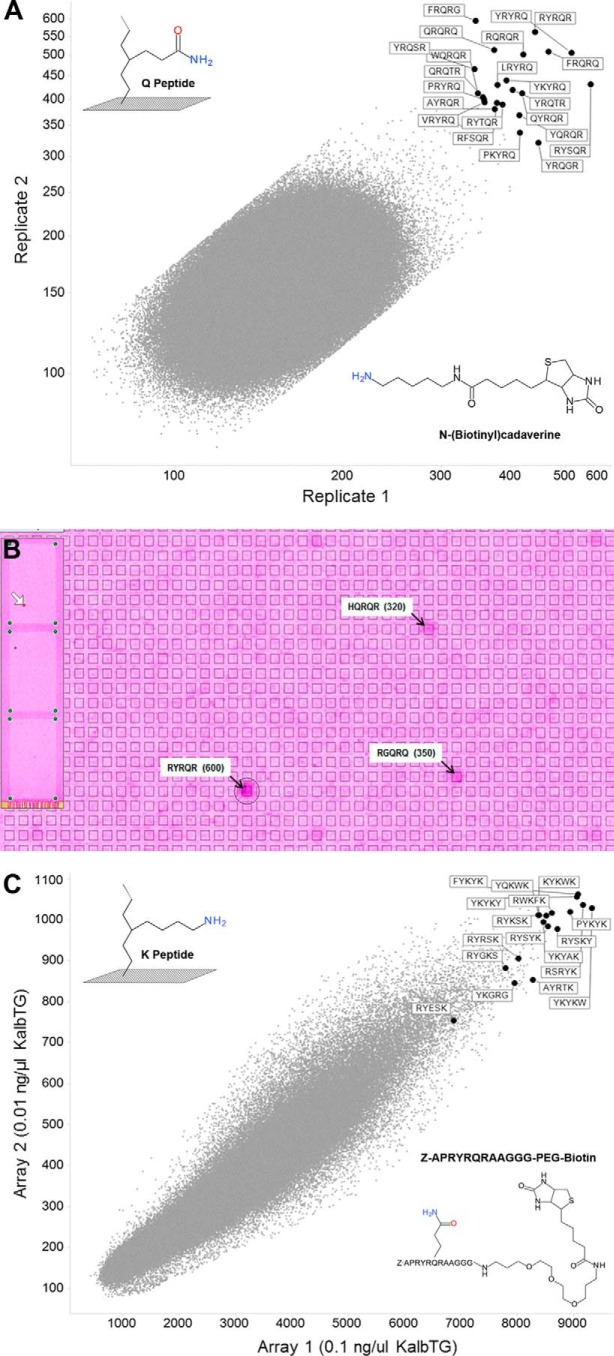
**KalbTG substrate discovery.** *A*, Gln substrate discovery. Fluorescence signal generated by KalbTG in the presence of biotinylated amine-donor substrate. Substrate structures are shown schematically, and the TG-reactive groups are *colored*. The plot shows correlation between two replicates of 1.4 million unique 5-mer peptides. 22 peptides with highest average fluorescence signal are tagged by their respective 5-mer peptide sequence. *B*, image analysis of KalbTG Gln substrate array (*A*). *Left*, image of the whole array containing 2,721,464 features (*spots*) with 5-mer peptides synthesized in a *chessboard pattern*. The *white arrow* shows position of one peptide of the RYRQR pair. The *main panel* shows an *enlarged area* around the RYRQR feature. RYRQR (*circled*) and two other peptides with detectable KalbTG activity are marked by *black arrows* with fluorescent signal shown in *parenthesis*. The average background signal over the array is ∼180. *C*, K-substrate discovery. Fluorescence signal generated on 5-mer array in the presence of KalbTG and biotinylated Gln donor substrate (Z-APRYRQRAAGGG-PEG-biotin). Substrate structures are shown schematically, and the TG-reactive groups are *colored*. Data are plotted as a correlation between average signal for unique 5-mer Lys peptides generated on two independent arrays with 0.1 ng/μl (*x* axis) or 0.01 ng/μl (*y* axis) KalbTG, respectively. Tagged by respective 5-mer sequence are 11 peptides with the highest average fluorescence signal and six single-Lys peptides tested in the GLDH-coupled assay. The data were filtered to remove noise as described under “Experimental procedures.”

**Figure 2.**
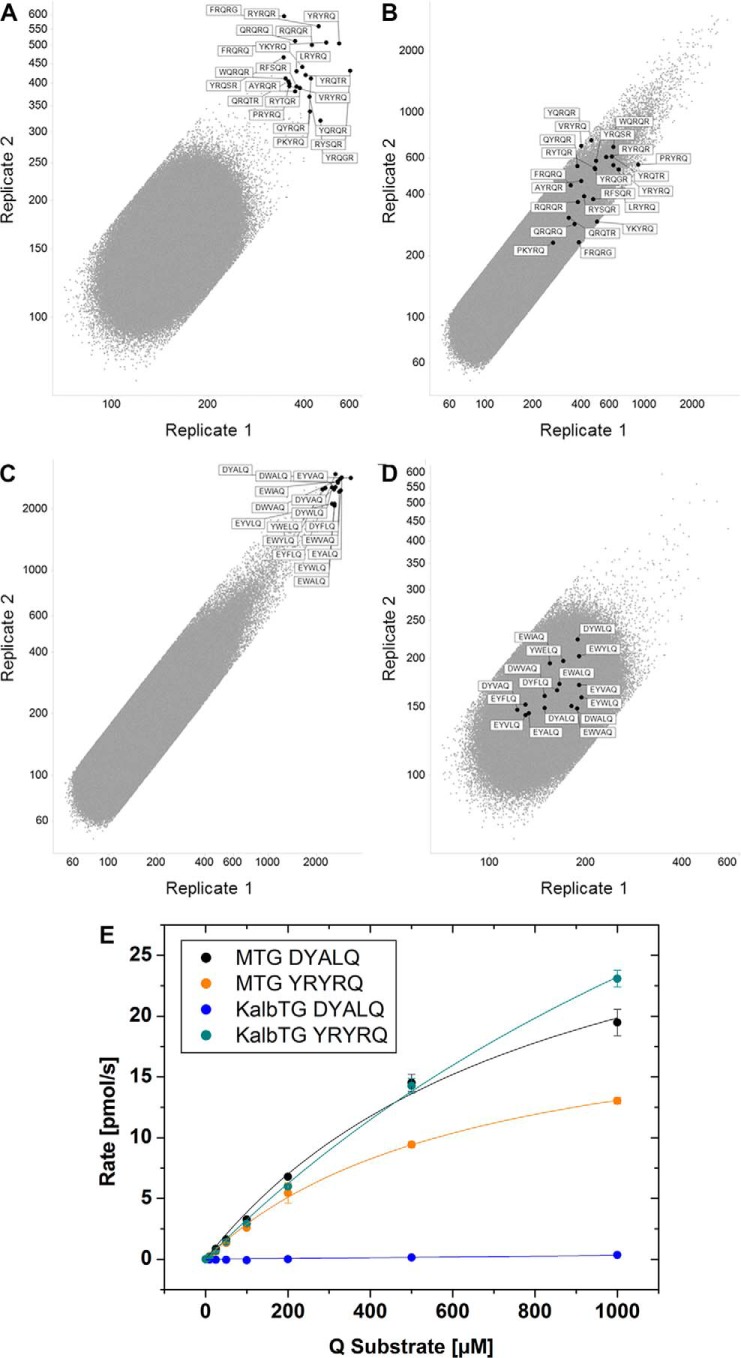
**Array cross-reactivity of KalbTG with MTG substrates.** *A*, correlation of fluorescence signal between two replicates, generated by KalbTG on a 2.8-million 5-mer array in the presence of biotinylated amine-donor substrate. 22 peptides with the highest signal intensity are highlighted within the plot. *B*, correlation plot of fluorescence signal generated by MTG on a 5-mer array in the presence of biotinylated amine-donor substrate. The fluorescence signals of the 22 peptides that exhibited the highest signals in the KalbTG array (*A*) are highlighted within the plot. *C*, plot of fluorescence signal generated by MTG on a 5-mer array in the presence of biotinylated amine-donor substrate. The 16 highest fluorescence signals are tagged by their respective 5-mer peptide sequence. *D*, plot of fluorescence signal generated by KalbTG on a 5-mer array in the presence of biotinylated amine-donor substrate. The fluorescence signals of the 16 peptides that exhibited the highest signals in the MTG array are highlighted within the plot. The average background signal over the array is ∼180. *E*, plots of MTG and KalbTG activity obtained by measuring rates of NADH oxidation at 340 nm and 37 °C using various concentrations of Z-GGGDYALQGGGG (0–1 mm) and Z-GGGYRYRQGGGG (0–1 mm) in the presence of amine-donor substrate cadaverine (1 mm) in the GLDH-coupled assay. *Error bars*, S.D.

The experiments were performed on two arrays in parallel, and the sequences of the peptides with the highest turnovers were determined (Fig. 1, *A* and *B*). The 9 best peptides were resynthesized and tested for KalbTG activity in a stand-alone glutamate dehydrogenase (GLDH)-coupled assay (supplemental Table 1). The assay was specifically optimized for the analysis of potential high-affinity peptides and utilizes the ammonia released in the transglutaminase reaction as a substrate in the GLDH-catalyzed and NADH-dependent reductive amination of α-ketoglutarate (21). This confirmed YRYRQ and RYRQR as the best 5-mer substrates (KalbTG Q-tag), with turnover rates of 3.52 ± 0.08 pmol of NADH/s and 3.60 ± 0.12 pmol of NADH/s, respectively. Lys-containing substrate YKYRQ exhibited the highest rates in the GLDH assay (4.00 ± 0.18 pmol/s NADH) but showed a relatively low turnover on the peptide array. We suspect that this may be an artifact caused by Lys cross-reactivity in solution and thus omitted this motif in further analysis. Surprisingly, no activity could be detected with the well-known MTG recognition motif MLAQGS (22) or the MTG substrate DYALQ discovered on our peptide array (Fig. 2 and supplemental Table 1).

A second round of maturation on the array yielded APRYRQRAA as the best 9-mer substrate, which was then resynthesized as biotinylated peptide to act as acyl donor for the discovery of optimized Lys recognition motifs (KalbTG K-tag) back on the 5-mer peptide array (Fig. 1*C*). Again, six of the best Lys peptides from the array were resynthesized and tested in the GLDH-coupled assay, now using a peptide containing the optimized Gln recognition sequence YRYRQ as acyl donor (supplemental Table 1). The highest turnover (4.47 ± 0.16 pmol NADH/s) in the GLDH assay was observed with the sequence RYESK.

### KalbTG is highly specific for matured glutamine substrates and enables efficient applications for bio-orthogonal conjugation

Because the peptide array can deliver readout for a comprehensive set of 5-mer peptides at once, a single data set each suffices to evaluate how enzymes differ in substrate specificity. The top KalbTG Gln substrates (Fig. 2*A*) can thus be found in the midfield of the signal distribution on the array performed with MTG (Fig. 2*B*), and, vice versa, the best-performing MTG Gln substrates (Fig. 2*C*) exhibit only signal close to background level on the KalbTG array (Fig. 2*D* and supplemental Table 1). To confirm that the two transglutaminase enzymes have orthogonal Gln substrate preferences and to quantify the amount of cross-reactivity, we determined the kinetics of both enzymes in the presence of varying concentrations of Z-GGGYRYRQGGGG and Z-GGGDYALQGGGG substrate peptides (Fig. 2*E*). MTG exhibited similar *K_m_* values in the 0.6–0.9 mm range for both substrates, whereas *k*_cat_ was significantly higher with the preferred DYALQ substrate (1.39 s^−1^ *versus* 0.93 s^−1^ with YRYRQ), resulting in catalytic efficiencies (*k*_cat_/*K_m_*) of 1.64 × 10^3^ and 1.44 × 10^3^ m^−1^ s^−1^, respectively (Table 1). Compared with the engineered MTG enzyme from Zedira, KalbTG appears to have a lower substrate binding efficiency (*K_m_* of 2 mm) but higher turnover (*k*_cat_ of 1.92 s^−1^), leading to *k*_cat_/*K_m_* of 0.89 × 10^3^ m^−1^ s^−1^. KalbTG appeared to be completely unreactive toward MTG substrate Z-GGGDYALQGGGG; thus, no kinetic parameters could be determined.

**Table 1:** **KalbTG/MTG cross-reactivity and kinetic parameters**

Parameters	MTG DYALQ	MTG YRYRQ	KalbTG DYALQ	KalbTG YRYRQ
*V*_max_ (pmol/s)	37	22	ND*^a^*	74
*K_m_* (μm)	847	644	ND	2151
*k*_cat_ (s^−1^)	1.39	0.93	ND	1.92
*k*_cat_/*K_m_* (m^−1^ s^−1^)	1640	1440	ND	890

*^a^* ND, not detectable.

**Figure 3.**
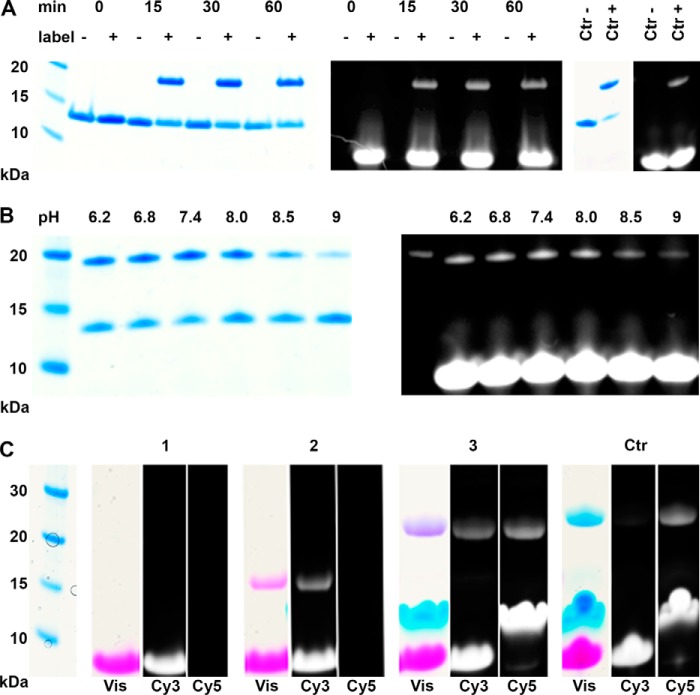
**Site-specific and bio-orthogonal conjugation with KalbTG.** *A*, time course of Cy3 labeling of a Q-tagged *T. thermophilus* SlyD moiety. Successful monolabeling is observed by the shift in electrophoretic mobility corresponding to the 5.9-kDa molecular mass of the label and by the fluorescent signal of the labeled species. A 0–60 min time course was performed with 10-fold label excess and controls without label addition. *Ctr*− and *Ctr*+, control reactions with SlyD containing the MTG Q-tag (DYALQ) and KalbTG or MTG, respectively. *B*, pH profile of KalbTG labeling efficacy. The highest labeling yield after 15 min of reaction time is observed at pH 7.4. *C*, dual site-specific functionalization of the construct YRYRQ-PEG27-Xa cleavage site-PEG27-PEG27-DYALQ (6.6 kDa) with two fluorophores; a mixture of peptide construct and a 10-fold excess of KalbTG K-tag-Cy3 label (*1*, 5.9 kDa) is incubated with KalbTG enzyme for 30 min to afford the monolabeled construct (*2*, 12.5 kDa). Then, with no intermediate blocking or purification steps, MTG enzyme and a 10-fold excess of MTG K-tag-Cy5 label (5.7 kDa) are added, and the mixture is incubated for 15 min to afford the dually labeled construct (*3*, 18.2 kDa). When changing the reacting order by first incubating the peptide construct and the 10-fold excess of each label with MTG for 15 min, a dually, mostly MTG K-tag-Cy5-labeled construct is afforded (*Ctr*, 18 kDa). Note that low electrophoretic mobility of the cyanine labels leads to higher apparent molecular weights compared with the protein marker.

Next, we applied the array and in-solution data to perform site-specific labeling on protein substrates. The molecular chaperone SlyD is an ideal scaffold for labeling approaches because epitope-containing loops can be grafted onto the FKBP-type domain, which optimally presents them to binders or enzymes (23). We produced a chimeric protein consisting of the *Thermus thermophilus* FKBP-type domain and the KalbTG recognition sequence RYRQR. Labeling with a 10-fold excess of KalbTG K-tag-Cy3 and a substrate/enzyme ratio of 72:1 afforded ∼70% yield of a labeled protein species after 15 min (Fig. 3*A*). This yield remained constant over a time course of 60 min. The molecular mass shift from 13 to 19 kDa was observed by SDS-PAGE, corresponding exactly to the incorporation of a single 6-kDa label molecule. An identically constructed FKBP-type domain, containing the MTG sequence DYALQ instead of RYRQR, showed no incorporation of label when incubated with KalbTG (Fig. 3*A*), signifying that the reaction is limited to the site of the KalbTG recognition motif and that none of the five other glutamines intrinsic to the FKBP-type domain are recognized. We furthermore assayed the pH dependence of the labeling reaction at pH 6.2, 6.8, 7.4, 8.0, 8.5, and 9 (Fig. 3*B*). The highest labeling efficiency after 15 min was found at pH 7.4, with activity trailing off at pH ≥8.5. These findings correspond well to the published pH preferences of MTG (15).

We used the high sequence specificity of KalbTG to conjugate a 6-kDa KalbTG K-tag-Cy3 label to the YRYRQ site of a 7-kDa substrate peptide comprising both the KalbTG and MTG Gln motifs. The reaction was run for 30 min to saturate the YRYRQ site. Analysis by SDS-PAGE confirmed that the label was integrated at a single site (Fig. 3*C*, *lane 2*). The subsequent incubation for 15 min with MTG and a 6-kDa MTG K-tag-Cy5 label in the same reaction vessel resulted in the formation of a dually labeled conjugate in high yield, with nearly all single-labeled species having visibly been converted to dually labeled (Fig. 3*C*, *lane 3*).

**Figure 4.**
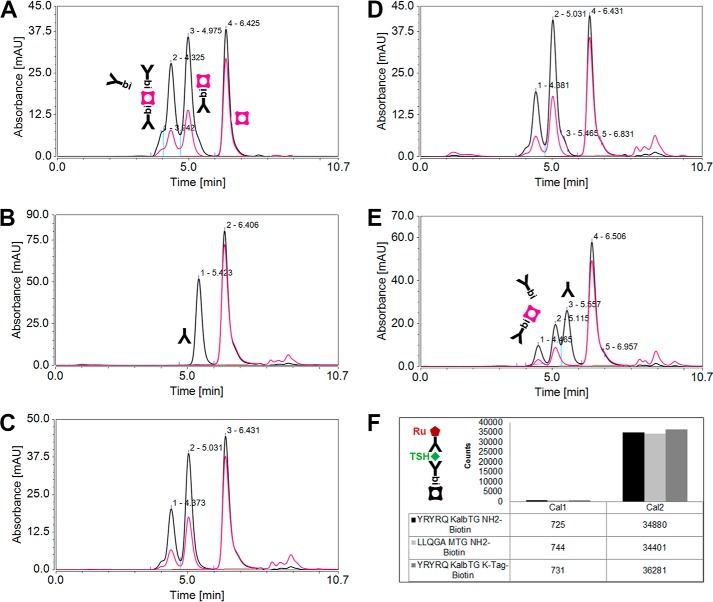
**Enzymatic biotinylation by MTG and KalbTG and functional characterization of anti-TSH IgGs tagged C-terminally either with LLQGA or YRYRQ motifs.** Shown are size-exclusion chromatography profiles of SA-FLUO–IgG-biotin complexes and unbound antibodies. *Black chromatograms*, protein absorbance (280 nm); *red chromatograms*, fluorescein absorbance (494 nm). *A*, SA-FLUO analytics of LLQGA-tagged antibody (TU1.20), labeled with MTG and a 100-fold excess of biotin-dPEG(23)-NH_2_. *Peaks 2*, *3*, and *4*, SA-FLUO molecules complexed with two, one, or no biotinylated IgGs, respectively. *Peak 1*, larger, undefined SA-FLUO–IgG complexes. *B*, SA-FLUO analytics of LLQGA-tagged antibody (TU1.20), incubated with KalbTG and a 100-fold excess of biotin-dPEG(23)-NH_2_. *Peaks 1* and *2*, unbiotinylated IgG and unbound SA-FLUO, respectively. *C*, SA-FLUO analytics of YRYRQGGS-tagged antibody (TU1.20), labeled with KalbTG and a 25-fold excess of biotin-dPEG(23)-NH_2_. *Peaks 1*, *2*, and *3*, SA-FLUO molecules complexed with two, one, or no biotinylated IgGs, respectively. *D* and *E*, enhanced selectivity of KalbTG for K-tag amino donors. SA-FLUO analytics of YRYRQGGS-tagged antibodies (TU1.20) conjugated by KalbTG with a 5-fold excess of either K-tag-biotin label (*D*) or biotin-dPEG(23)-NH_2_ label (*E*). *Peaks 1*, *2*, and *4*, SA-FLUO molecules complexed with two, one, or no biotinylated IgGs, respectively. *Peak 3*, unbiotinylated IgG. *F*, performance of the purified IgG-biotin conjugates from *A* (LLQGA MTG NH_2_-biotin), *C* (YRYRQ KalbTG NH_2_-biotin), and *D* (YRYRQ KalbTG K-tag-biotin) in the TSH Elecsys immunoassay. The TSH sandwich assay principle is shown schematically.

As an example for labeling of antibodies for use in therapeutic and immunodiagnostic applications, we inserted the KalbTG Q-tag to the heavy chain C terminus of the IgG used in the Elecsys TSH electrochemiluminescence immunoassay. IgG biotinylation via KalbTG was assessed by complex formation with fluorescein-labeled streptavidin (SA-FLUO). Successful complexation (IgG-Bi–SA-FLUO and IgG-Bi–SA-FLUO–Bi-IgG) is observed as a fluorescent double peak in the elution profile of an analytical size-exclusion chromatography (Fig. 4*C*). As a control, the same IgG modified C-terminally with the MTG Q-tag LLQGA published by Rinat-Pfizer Inc. (7) was biotinylated via MTG. It exhibited a similar pattern in the SA-FLUO analytics (Fig. 4*A*); however, compared with the KalbTG experiment, a higher excess of biotin label and higher enzyme/IgG ratio was needed to achieve this result. When incubated with KalbTG, the LLQGA-tagged IgG showed no SA-FLUO complex formation. Instead, a single absorbance peak of the unmodified IgG was observed (Fig. 4*B*). When further reducing the biotin label excess, it became apparent that incorporating the array-discovered KalbTG K-tag peptide in the biotin label adds another level of specificity to the reaction; IgG was almost completely covalently modified when using a 5-fold molar excess (equaling 2.5-fold excess/tagged heavy chain) of K-tag-biotin label (Fig. 4*D*), whereas only partial modification was observed when using a commercially available biotin-dPEG(23)-NH_2_ label in 5-fold excess (Fig. 4*E*). Presumably, a significant portion of the Q-tag glutamine side chain is hydrolyzed by the transglutaminase in the absence of a suitable amine donor. The degrees of IgG biotin modification were confirmed by mass spectrometry (data not shown). Finally, the viability of biotin-modified IgG was tested in the Elecsys TSH (sandwich) assay. The chemically biotinylated capturing antibody in the R1 compartment of the original reagent pack was replaced with the IgG biotinylated via MTG or KalbTG. Both antibodies performed comparably to the original antibody of the commercial assay (Fig. 4*F*).

### The compact crystal structure of KalbTG suggests a peptide substrate binding mode

**Figure 5.**
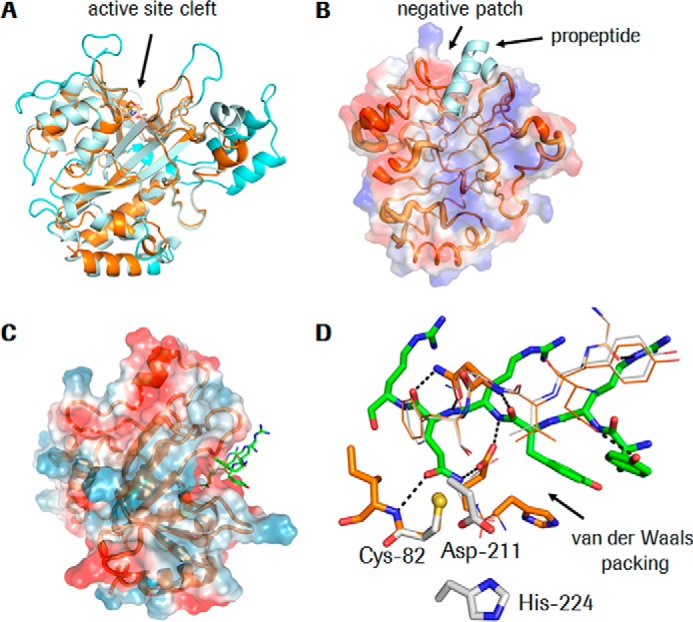
**Structural characterization of KalbTG.** *A*, KalbTG is shown as an *orange ribbon*, and *S. mobaraensis* MTG is shown in *light blue* with the longer loops (compared with KalbTG) *colored* in *cyan*, highlighting the much more compact structure of KalbTG. *B*, the surface potential of KalbTG shows a negatively charged area next to the substrate and propeptide binding site. The propeptide of *S. mobaraensis* MTG (kinked and helical) is placed at the equivalent position of KalbTG to highlight the overall similar shape of this pocket. The backbone trace of KalbTG varies in thickness as a function of *B*-values, indicating that loop regions further away from the active site are more flexible than the area around the active site, which is not involved in crystal contacts. *C*, model of the extended sequence YRYRQR (*green*) in the active site. The Gln side chain is in close vicinity to the catalytic Cys-82. The positively charged peptide is steered to the negative patch at the active site. *D*, detailed view of the peptide model (*green sticks*) from *C*. The starting KalbTG structure is shown in *gray*, and the model after energy minimization is shown in *orange*, with key residues interacting with the peptide highlighted in *thick sticks*. A number of possible hydrogen bonds are drawn as *dashed black lines*. The peptide and its surrounding residues were energy-minimized in MOLOC (www.moloc.ch)^3^ (54) while keeping the remainder of KalbTG fixed. Minor movements of KalbTG are required to adapt the substrate binding site to the peptide. Figures were prepared with PyMOL.

To gain further insight into how the small KalbTG sequence can fold into such an efficient MTG and whether the substrate specificity as determined by the peptide array can be explained, we determined the crystal structure of KalbTG to a resolution of 1.9 Å. Superposition of KalbTG with its next closest homolog, the MTG from *S. mobaraensis* (24), shows a similar disc-shaped core structure (root mean square deviation of 1.5 Å) of a central β-sheet with flanking α-helices and a surface depression forming the active site cleft. However, KalbTG is more compact, having much shorter surface loops (Fig. 5*A*). In fact, KalbTG is the smallest MTG reported to date, even smaller by 2 kDa than the structurally unrelated *Bacillus subtilis* TG (25)(PDB entry 4P8I; 245 amino acids, 28.3 kDa), showing that the same degree of structural economy for MTGs can be reached by convergent evolution. Thermophilic proteins often have short surface loops, which reduce the entropy of the protein, and this property might also contribute to the high affinity of KalbTG.

The catalytic Cys-Asp-His triad of KalbTG is located at the bottom of the active site groove (Fig. 5*A*). The groove is wide enough to be covered by a kinked helical propeptide in the unprocessed enzyme (Fig. 5*B*), similar to what has been observed with the *S. mobaraensis* MTG zymogen (26), indicating that a similar zymogenic mechanism may be present in KalbTG. Strong difference electron density extending from Cγ of the catalytic Cys-82 (supplemental Fig. 3) showed that this residue is modified by a thiol-reactive compound, the origin of which is unknown. The electron density allows tracing of four atoms, and these were tentatively modeled as a β-mercaptoethanol disulfide adduct of Cys-82. The isosteric cysteamine or a larger compound that is disordered beyond the fourth atom cannot be excluded. A modified Cys in the active site of MTGs is also apparent when inspecting the electron density maps for Cys-110 in *S. mobaraensis* (PDB entry 3IU0), Cys-116 in *B. subtilis* (PDB entry 4PA5; modeled as cysteamine adduct in chain A), and Cys-302 in *Streptococcus suis* (PDB entry 4XZ7; adduct not modeled for one molecule in the asymmetric unit and as a string of water molecules in the second molecule). As in all other transglutaminase structures, the catalytic Cys-82 in KalbTG is located at the N terminus of an α-helix, which reduces its p*K_a_* and increases its nucleophilicity (supplemental Fig. 4) for attack on the substrate glutamine. Of note, the catalytic Cys-82 in KalbTG is embedded 1.7 Å deeper in the active cleft than its *S. mobaraensis* MTG counterpart, raising the question of how the Gln side chain of the substrate peptide might reach it.

Attempts to model the sequence YRYRQR in α-helical conformation, guided by the structure of the propeptide (PDB entry 3IU0; Fig. 5*B*) were unsuccessful because the Gln side chain would not come close enough for the required attack by Cys-82. By contrast, an extended, β-strand conformation of the peptide can be placed into the active site of KalbTG with no steric repulsions and several possible hydrogen bonds between peptide and KalbTG (Fig. 5*C*). In this conformation, the Arg side chains would all point away from KalbTG, indicating that negative potential on KalbTG might help in electrostatic steering of positively charged peptides to the active site. This assumption is supported by the fact that both Lys and Arg are prominent in the array-discovered substrate peptides (supplemental Table 1). In the model, the second Tyr of the YRYRQR sequence packs on the His-188 side chain of KalbTG. Other active peptide sequences found in the peptide array have Phe and Gln at this position, which can entertain similar hydrophobic interactions. By contrast, the inactive peptides DYALQ and MLAQG have Ala and Leu, respectively, at this position, which would engage in fewer van der Waals interactions with His-188. A structure of a KalbTG–peptide complex will be required to verify the proposed binding mode. However, initial co-crystallization and soaking experiments to this extent have so far been unsuccessful.

## Discussion

We describe the design, production, and structural characterization of a novel microbial transglutaminase KalbTG as well as the high-throughput screening of substrate peptides via an ultra-dense peptide array approach. The array-determined substrate sequences were further used, together with MTG and its preferred substrates, for orthogonal conjugation of biomolecules.

Establishing a viable and robust enzymatic industrial scale method for site-specific conjugation approaches, such as antibody-drug conjugates, poses high demands on the coupling enzyme; it requires a high catalytic efficiency and substrate specificity and has to be economical to produce, preferably of low molecular mass, and independent of cofactors. These requirements are met by KalbTG; it is as efficient as previously known MTGs but exhibits increased specificity and developability. Furthermore, the enzyme requires no additives and works well in standard buffers, such as Tris, MOPS, or PBS.

Site-specific labeling with a promiscuous enzyme can be the method of choice if (*a*) the substrate is not recombinantly modified with a conjugation tag, in which case naturally occurring residues are used, and (*b*) the labeling site and label ratio can be controlled or are not of critical importance for the application at hand. Examples of this type of application are the conjugation of payloads to deglycosylated/glycosylated IgG (9), biotinylation of antibodies via glutamine residues (27), or using the Gln-295 within the heavy chain of IgGs as a substrate (28). However, using a nonspecific enzyme severely limits the range of possible applications (29) and may lead to unwanted side reactions, such as cross-linking of IgGs. Our approach combines an enzyme with naturally high substrate specificity, KalbTG, with an effective substrate screening for recombinant tags.

Biotechnological optimization and industry-scale recombinant production of MTGs are difficult, as illustrated by a plethora of recent publications (30–39). Genetic modification of the pro-domain residues Tyr-14, Asp-20, Ile-24, and Asn-25 has been reported to increase production levels of soluble and active MTG in *E. coli*. These modifications maintain tight pro-domain interaction with the enzyme to avoid the inherent cross-linking activity of MTG that is toxic to the host cell (40) and also complicates *in vitro* handling. Overall, the MTG production processes involving the inactive pro-form have not fundamentally changed since they were originally described (16).

In our study, we found no evidence of the predicted propeptide sequence TTAQAAAVAAPTPR to bind or block the enzyme. Because the predicted sequence may not comprise the whole propeptide, a zymogen-activation approach for KalbTG would require significant effort. However, we found that the addition of NH_4_^+^, the product of the transglutaminase reaction, strongly inhibits activity of KalbTG. This simple step increased the yield and facilitated downstream processing, crucially enabling us to produce soluble transglutaminase without a specific pro-sequence and activating it by a simple dialysis step. This represents a marked improvement over any published MTG production process to date.

Peptide arrays can be highly efficient for high throughput enzyme characterization (12, 41). In this study, we synthesized arrays with millions of spatially addressable peptides using a light-directed, digitally controlled process and developed methods for *in situ* analysis of enzyme activity and substrate specificity for both KalbTG and MTG. Importantly, this screen allowed selection of transglutaminase substrate peptides to facilitate an orthogonal labeling with enzymes formerly known as being highly promiscuous. We consider ultra-high-density peptide arrays to be an enabling technology that will contribute to a wide field of enzyme/substrate research, including transferases, ligases, and proteases.

Whereas the compact structure of KalbTG with its comparatively shorter surface loops should have lower entropy and thus higher catalytic efficiency, this is not observed when comparing the activities of MTG and KalbTG (Table 1), indicating the presence of mutually compensating effects. However, the smaller KalbTG is an advantage for biotechnological production. Whereas in other MTGs, a helical pro-peptide is necessary to physically block the active site and avoid detrimental activity (26), elevated concentrations of NH_4_^+^ are sufficient to block KalbTG activity as long as it is required. This simple mechanism avoids the biotechnologically tedious step of proteolytic maturation. The negative surface potential close to the active site might constitute a binding site for inhibitory NH_4_^+^. However, these are difficult to distinguish from water in electron density maps, and no NH_4_^+^ has been identified in the KalbTG structure based on geometric criteria (four hydrogen bond acceptors in a tetrahedral geometry). Interestingly, we found the sequence YRYRAR, inherent in a KalbTG surface loop, to closely resemble our preferred array-discovered Gln substrate peptides. However, rigidity and position of the loop prevent its interaction with the active site, therefore raising the question of what role it may play in substrate recruitment.

The KalbTG structure and the peptide model set a starting point for rational engineering of further improved or altered substrate specificity. In combination with peptide array-based high-throughput substrate screening, this will enable the creation of tailor-made enzyme–substrate pairs with extremely versatile and orthogonal uses.

Our dual-labeling experiments confirm that KalbTG and MTG constitute an orthogonal conjugation system with unparalleled ease of use, yield, and efficiency. Furthermore, as exemplified in the antibody conjugation experiments and TSH Elecsys immunoassay, KalbTG enables true site-specific labeling with minimal reaction and reagent requirements, making it highly attractive for the industrial-scale synthesis of complex protein conjugates of interest in therapeutic or diagnostic applications.

An important question that naturally arises when an artificial peptide sequence is introduced into a molecule intended for potential therapeutic use is whether it may provoke immunogenic reactions. A peptide search on Proteome database UniprotKB yielded 3,985 matches of the pentapeptide YRYRQ, of which a single one is in *Homo sapiens*. A substring search of both YRYRQ and RYESK in the Immune Epitope Database (http://www.iedb.org)^3^ (53) yielded no results. As a comparison, the previously published MTG Q-tag LLQGA (2) yielded 55,145 matches in UniprotKB, 360 of which are found in human proteins. Six records of immunogenic epitopes containing the substring were found.

The introduced Q-tag peptide sequence described here can therefore be seen as a relatively rare motif in nature, putatively less prone to elicit immune responses by cross-reactive neutralizing antibodies to endogenous proteins. Furthermore, allowing specific single-amino acid substitutions in the tag sequences may strongly modulate potential immunogenicity while retaining decent transglutaminase reactivity.

The immune response (one factor being HLA/MHC reactivity) to protein therapeutics is complex and difficult to predict, leaving the “immunogenicity question” a hot topic in antibody-drug conjugate development to be closely monitored in future studies (42).

## Experimental procedures

### Bioinformatic methods

The web interface of NCBI Protein BLAST (43) was used to search for sequences similar to the MTG of *S. mobaraensis*. The amino acid sequence of *S. mobaraensis* protein–glutamine γ-glutamyltransferase (Uniprot accession number P81453) was entered as a query. Manual screening of the results for *E* values < 10^−10^ and polypeptide sequences shorter than that of *S. mobaraensis* MTG yielded hypothetical gene product KALB_7456 from bacterial strain *K. albida* DSM 43870 (GenBank^TM^ accession number AHI00814.1, Uniprot accession number W5WHY8). Sequence alignment of the *S. mobaraensis* and the *K. albida* sequences with Clustal Omega version 1.2.1 (44) yielded a value of 32% in the percent identity matrix and identified conservation of the catalytically active residues of MTG (Cys-140, Asp-331, and His-350; P81453 numbering). The ProP 1.0 server from the Technical University of Denmark (45) was used to predict the propeptide and signal sequences of the hypothetical *K. albida* microbial transglutaminase. VAAPTPR↓AP was the only predicted propeptide cleavage site with a score (0.513) above the threshold.

### Production of KalbTG

The gene sequence encoding for hypothetical *K. albida* microbial transglutaminase was codon-optimized for *E. coli* expression (Roche Sequence Analysis Web interface), chemically synthesized (GeneArt, ThermoFisher, Regensburg), and cloned via fragment exchange cloning (19) into a vector, conferring two N-terminal moieties of sensitive-to-lysis D chaperones (SlyD, Uniprot entry P0A9K9), truncated after Asp-165 (20), followed by a protease factor Xa cleavage site and including a C-terminal octa-His tag. The vector is based on the pQE-80 series by Qiagen, including isopropyl 1-thio-β-d-galactopyranoside-inducible protein expression by T5 promotor and conferring resistance to ampicillin. Plasmid preparation and transformation of chemically competent *E. coli* Bl21 Tuner cells with the expression plasmid were performed according to standard molecular biology protocols (46).

Fermentation was carried out at 35 °C for 26 h, until an *A*_600_ of 44 was reached. Cells were harvested and resuspended in buffer containing 50 mm Tris-HCl, pH 8.0, 1 mm EDTA, 1 mm DTT, and 10 mm (NH_4_)_2_SO_4_. Cells were disrupted by a high-pressure homogenizer at 800 bars. The resulting cellular extract was pretreated with 1–3% Polymin-G20 and then loaded onto a Q-Sepharose XL column (strong anion exchange matrix; GE Healthcare Life Sciences) at a protein concentration of ∼30 mg/ml. Bound protein was washed with 20 mm Tris-HCl, pH 8.0, 1 mm EDTA, 1 mm DTT, 10 mm (NH_4_)_2_SO_4_, and 150 mm NaCl and then eluted with a 30-column volume gradient from 150 to 500 mm NaCl. The eluate was dialyzed (10,000 molecular weight cutoff) against 20 mm Tris-HCl, pH 8.0, 0.1 mm EDTA, 0.1 mm DTT, 10 mm (NH_4_)_2_SO_4_, 500 mm NaCl; concentrated; and loaded onto a nickel-nitrilotriacetic acid column. Bound, His-tagged protein was washed with 20 mm Tris-HCl, pH 8.0, 0.1 mm EDTA, 0.1 mm DTT, 10 mm (NH_4_)_2_SO_4_, 500 mm NaCl, 25 mm imidazole and eluted with a 20-column volume gradient from 25 to 200 mm imidazole. Purified protein was dialyzed (10,000 molecular weight cutoff) against 20 mm Tris-HCl, 1 mm EDTA, 1 mm DTT, and 10 mm (NH_4_)_2_SO_4_, pH 8.0; concentrated to 1.77 mg/ml; analyzed by an SDS-PAGE and GLDH activity assay; and frozen in 10-mg aliquots at −80 °C. Prior to use, (NH_4_)_2_SO_4_ was removed by dialysis with a 10-kDa molecular weight cutoff filter to yield the active enzyme. For some applications, Factor Xa proteolysis was performed to remove the 2× SlyD portion from the KalbTG construct.

### Peptide array

A library of 1,360,732 unique 5-mer peptides was designed by using all combinations of 18 natural amino acids, excluding cysteine and methionine as well as any dimer or a longer repeat of the same amino acid and any peptide containing HR, RH, HK, KH, RK, KR, HP, and PQ sequences. The library was synthesized in duplicate on the same array by using maskless light-directed peptide array synthesis (Roche Nimblegen). Each 5-mer peptide was flanked from both the N and C terminus by 3-amino acid linkers synthesized by using a mixture of Gly and Ser at a 3:1 ratio. Peptide synthesis was accomplished through light-directed array synthesis in a Roche NimbleGen maskless array synthesizer using an amino-functionalized substrate as reported previously (13). Prior to use, arrays were incubated in blocking buffer (10 mm Tris-HCl, pH 7.4, 1% alkali-soluble casein (EMD Chemicals), 0.05% Tween 20) at room temperature for 1 h.

To test KalbTG specificity for Gln substrate, *N*-(biotinyl)cadaverine (Zedira) was used as a substitute for a Lys substrate to biotinylate Gln peptides on a peptide array. A KalbTG-labeling reaction was performed in 1,200 μl of 100 mm Tris-HCl, pH 8, 1 mm DTT, 50 μm *N*-(biotinyl)cadaverine, 0.2 ng/μl KalbTG in a SecureSeal^TM^ chamber (Grace Bio-Labs) at 37 °C for 45 min. After incubation, the chamber was removed, and the array was washed in 20 mm Tris-HCl, pH 7.8, 0.2 m NaCl, 1% SDS for 1 min, followed by a 1-min wash in 20 mm Tris-HCl. Biotin linked to the array was stained with 0.3 μg/ml CyTM5–streptavidin (GE Healthcare) in blocking buffer at room temperature for 1 h. Cy5 fluorescence intensity was measured with a MS200 scanner (Roche Nimblegen) at a resolution of 2 μm and wavelength of 635 nm.

Because array synthesis is digitally programmed, a new array can be designed and synthesized in a matter of few days. This allowed us to quickly verify and mature sequence motifs selected with the 5-mer arrays. In the case of maturation of the APRYRQRAA peptide, the new array design was created by extending the YRYRQ and RYRQR 5-mer sequences with all possible combinations of 2 amino acids from both the N and C terminus (*i.e.* each 5-mer sequence was extended to 160,000 9-mers with an invariant core motif). 9-Mers with the highest KalbTG activity on array were used to design another new array that included all possible single and double substitutions of selected 9-mers with all 20 natural amino acids. This step allowed us to “mutate” the original 5-mer core sequence and evaluate restrictions imposed by the 5-mer array peptide selection.

To test KalbTG specificity for Lys substrates, chemically synthesized Z-APRYRQRAAGGG-PEG-biotin peptide was used as a Gln substrate to biotinylate Lys peptides. Array biotinylation was done as described above with 0.01 or 0.1 ng/μl KalbTG and 0.8 μm peptide in 100 mm Tris-HCl, pH 8, 1 mm DTT, 0.05% Tween 20 at 37 °C for 15 min.

MTG reactions on the peptide array were performed in 100 mm Tris-HCl, pH 8, 1 mm DTT, 50% protein-free blocking buffer (Thermo Scientific) with 25 μm *N*-(biotinyl)cadaverine and 0.2 ng/μl MTG at 42 °C for 1 h.

Control experiments with CyTM5–streptavidin only were performed to show that no nonspecific streptavidin binding to the array occurred. Imperfections of the array surface typically result in a high signal intensity noise affecting ∼1–3% of peptide features. To remove noise, peptides for which |(*S*_1_ − *S*_2_)/(*S*_1_ + *S*_2_)| > 0.2 were excluded from analysis, where *S*_1_ and *S*_2_ represent signal intensity of replicates 1 and 2, respectively.

Because it is not technically possible to evaluate the yields and quality of each peptide on the array, a quality control process was developed where various peptides and their substitution variants that bind to streptavidin are synthesized on an array.^4^ The relative binding signals of streptavidin to these peptides is evaluated from array to array and is used to assess indirect quality of synthesis and overall performance of a synthesis run.

### GLDH-coupled assay

To determine whether the KalbTG peptides selected in the array assay were also preferred substrates in a solution reaction and to quantify cross-reactivity of KalbTG and MTG with various substrates, a continuous GLDH-coupled assay for MTG activity (21) was applied. For glutamine substrate evaluation, the assay was performed in a transparent 96-well microtiter plate in the presence of 500 μm α-ketoglutarate, 500 μm or 1 mm cadaverine as amine donor substituting for a Lys peptide, 2 units/ml of GLDH, 500 μm NADH, and Gln-containing substrate peptide (Z-GGGQRWRQGGGG, Z-GGGWRYRQGGGG, Z-GGGYRYRQGGGG, Z-GGGRYRQRGGGG, Z-GGGRYSQRGGGG, Z-GGGFRQRQGGGG, Z-GGGRQRQRGGGG, Z-GGGFRQRGGGGG, Z-GGGQRQRQGGGG, Z-GGGYKYRQGGGG, Z-GGGQYRQRGGGG, Z-GGGDYALQGGGG, or Z-GGGMLAQGSGGG) concentrations ranging between 0 and 1 mm in 200 mm MOPS, 1 mm EDTA, pH 7.2 (total volume per well 200 μl).

For amine substrate evaluation, assay conditions were identical, but 100 μm each of amine substrate (Z-GGGRYSKYGGGG, Z-GGGAYRTKGGGG, Z-GGGRYRSKGGGG, Z-GGGYKGRGGGGG, Z-GGGRYGKSGGGG, Z-GGGRYESKGGGG, Z-GGGPGRYKGGGG, Z-GGGARSKLGGGG, or cadaverine) and 200 μm Gln donor (Z-GGGYRYRQGGGG or Z-GGGDYALQGGGG) were used.

Reactions were started by the addition of 5 μg/ml MTG (Zedira) or KalbTG, and the oxidation of NADH was continuously recorded at 340 nm for 60 min using a Biotek Synergy H4 microplate reader, temperature-controlled at 37 °C, with short shaking intervals before each measurement. After a short lag phase where the GLDH was saturated by TG-mediated release of ammonia, linear rates of absorbance *versus* time, corresponding to TG turnover, were observed and subjected to Michaelis–Menten kinetic analysis. Rates of absorbance (milliabsorbance units/min) were converted into molar rates of NADH turnover (pmol/s) using the formula (previously determined by an NADH standard curve), turnover rate = ((|absorbance rate|) × 1.111).

### Labeling assays

The chaperone SlyD from *Thermus thermophilus* (Uniprot number Q5SLE7) was used as a labeling scaffold for KalbTG, by recombinant grafting of a KalbTG glutamine donor sequence (Q-tag) onto the FKBP-type domain, which yielded the polypeptide sequence MKVGQDKVVTIRYTLQVEGEVLDQGELSYLHGHRNLIPGLEEALEGREEGEAFQAHVPAEKAYGAGSGGGGRYRQRGGGGGSSGKDLDFQVEVVKVREATPEELLHGHAHHHHHHHH.

The protein was produced in *E. coli* Bl21 Tuner and purified by standard techniques (HisTrap, Superdex 200 pg).

Labeled peptides were chemically synthesized, to be composed of a Z-protecting group at the N-terminal amino group, a transglutaminase lysine donor sequence (K-tag) on the N-terminal sequence part, and a Cy3 or Cy5 fluorescent dye at the C terminus, spaced by a linker sequence.

The primary chemical structures were as follows: Z-RYESKG*O2Oc*EUEUEUEUEUEUEUEUEUEUEUEUEUEUEUEUEUEUEUEU*C(sCy3-MH)*-OH (KalbTG K-tag-Cy3, 5,863.9 g/mol) and Z-RSKLG*O2Oc*EUEUEUEUEUEUEUEUEUEUEUEUEUEUEUEUEUEUEUEU*C(Cy5-MH)*-OH (MTG K-tag-Cy5, 5,723.9 g/mol), where U is β-alanine, *O2Oc* is 8-amino-3,6-dioxaoctanoic acid, and *C(sCy3-MH)* and *C(Cy5-MH)* represent a C-terminal cysteine modified post-synthesis by sulfo-Cy3 maleimide and Cy5 maleimide, respectively.

For the orthogonal labeling experiment, a molecule containing both KalbTG and MTG Q-tags was chemically synthesized, with a primary chemical structure of Z-GGGYRYRQGGG*PEG27*GIEGRG*PEG27**PEG27*GGGDYALQGG-OH (6,620.6 g/mol).

All peptides were synthesized via standard Fmoc-based solid phase peptide synthesis in a 0.25-mmol scale using commercially available building blocks. After solid-phase synthesis, peptides were cleaved with TFA/triisopropylsilane/water (95:2.5:2.5) and precipitated with diisopropylether followed by purification via RP18-HPLC using a water/TFA acetonitrile gradient. Dye labeling was achieved by reaction of the peptides with sulfo-Cy3 maleimide (Lumiprobe) and sulfo-Cy5 maleimide (GE Healthcare), respectively. Purification of dye-labeled peptides was achieved by RP18-HPLC using a water/TFA acetonitrile gradient. Identity of the peptides was confirmed by LC-MS (Thermo Scientific RSLC-MSQplus system), applying a Kinetex C18 2.6-μm, 50 × 3-mm column (Phenomenex).

If not noted otherwise, labeling reactions were performed for 15 min at 37 °C in the presence of 72 μm substrate protein, 720 μm label peptide, and 1 μm transglutaminase in 200 mm MOPS, pH 7.2, and 1 mm EDTA. For the pH-dependent labeling profile, experiments were performed in 200 mm MOPS buffer adjusted to pH 6.2, 6.8, and 7.4 with NaOH or HCl or 200 mm Tris buffer adjusted to pH 8.0, 8.5, and 9.0 HCl. For the orthogonal labeling experiment, 1.5 μm KalbTG was added to a volume of 20 μl containing 100 μm substrate peptide and 1 mm KalbTG K-tag-Cy3. After incubation for 30 min at 37 °C, 1 mm MTG K-tag-Cy5 and 1.5 μm MTG were added and incubated for an additional 15 min at 37 °C. The reaction was stopped by the addition of 50 mm TCA. Samples were taken between incubation steps and analyzed by SDS-PAGE and in-gel fluorescence (Bio-Rad ChemiDoc gel documentation system, Cy3 and Cy5 LED, and filter sets).

### Crystallization and structure determination of KalbTG

KalbTG in PBS was crystallized at 22 °C using the sitting drop (200-nl) vapor diffusion method by 1:1 mixing of 8 mg/ml protein with an unbuffered reservoir consisting of 0.2 m ammonium tartrate, 20% PEG 3350. Crystals were cryoprotected in reservoir solution containing 20% ethylene glycol before flash-cooling in liquid nitrogen. Data were collected at 100 K at SLS beamline PX-II using a Pilatus 6M detector and integrated and scaled in space group P3 with XDS (47). The *l* = 3*n* reflections have *I*/σ of >9, rendering the presence of a screw axis unlikely. Self-Patterson and twinning analyses did not reveal suspicious data pathologies. The cell volume is consistent with two or three KalbTG molecules in the asymmetric unit, with Matthews parameters of 3.5 Å^3^/Da and 2.3 Å^3^/Da, respectively. Whereas the κ = 180° section of the self-rotation function did not indicate a 2-fold NCS axis, a peak in the κ = 164° section at ω = 0°, φ = 0° indicated that at least two molecules in the asymmetric unit are related by a 164° rotation, which turned out to be correct after molecular replacement. Data collection statistics are summarized in supplemental Table 1.

The structure of KalbTG (226 residues) was determined by molecular replacement using the *S. mobaraensis* transglutaminase (354 residues, PDB entry 3IU0) as the search model. The first attempts using the complete *S. mobaraensis* TG were unsuccessful, probably because the enzymes are of very different sizes. The two transglutaminases share 28.2% sequence identity and 38.9% sequence similarity over the entire length of KalbTG. A variant of *S. mobaraensis* TG devoid of loop regions and trimmed to the hydrophobic core resulted in a potential solution with PHASER (48) when searching for two molecules in the asymmetric unit in space group P3 with a log-likelihood gain of 213. Trigonal space groups P31 and P32 did not yield solutions, consistent with the high intensities of the *l* = 3*n* reflections. The molecular replacement model was refined in BUSTER (49) to an initial *R*_free_ of 46%. Some secondary structure elements were visible in the electron density maps and were included in the model, which was then submitted to 10 cycles of automatic model building and refinement in CBUCCANEER and REFMAC5 (50). The resulting model included the entire KalbTG catalytic domain and had an *R*_free_ of 30%. The structure was completed in COOT (51) and refined with PHENIX (52) to an *R*_free_ value of 23% at 1.9 Å resolution with excellent stereochemistry. There are two molecules in the asymmetric unit that are virtually identical (root mean square deviation 0.26 Å over all atoms) and exhibit excellent electron density (supplemental Fig. 3) and stereochemistry (supplemental Table 2). Model refinement statistics are collected in supplemental Table 2. The first 19 N-terminal amino acids (MGGGSTTAQAAAVAAPTPR) and the C-terminal artificial GGGS-His_8_ tag are disordered in the structure.

### DSC

Measurements were performed with a starting temperature of 20 °C and a final temperature of 90 °C on a VP-capillary DSC instrument (MicroCal/GE Healthcare) using PBS as a reference. A scanning rate of 90 °C/h was applied. The mature and active KalbTG enzyme with the 2× SlyD removed was measured at a protein concentration of 0.7 mg/ml in PBS. Data analysis was performed with Origin version 7, SW 2.0.

### Antibody expression and purification

Heavy and light chains of TU1.20 (mouse monoclonal antibody against TSH) were cloned into standard mammalian expression vectors featuring a CMV promotor (46) and including different conjugation tags (LLQGA and GGGSYRYRQGGGS) at the heavy chain C terminus. Both plasmids encoding heavy and light chain were co-transfected into suspension-adapted human embryonic kidney HEK293-F cells (Life Technologies/Thermo Fischer Scientific). HEK293-F cells were cultured in shaker flasks at 37 °C in FreeStyle 293 expression medium (Thermo Fisher Scientific) under serum-free medium conditions. The cells were transfected at ∼2 × 10^6^ vital cells/ml with the expression plasmids (0.5 mg/liter of cell culture) complexed by PEIpro (Polyplus) transfection reagent (1.3 ml/liter of cell culture) in PBS buffer. The culture supernatant was collected at day 7 post-transfection by centrifugation. IgG was purified via one-step protein A affinity purification (HiTrap MabSelect SuRe, GE Healthcare) according to the supplier's instructions.

### Biotinylation

TU1.20 tagged at the C terminus of each heavy chain with LLQGA (26.7 μm) was conjugated for 16 h at 37 °C with a 100-fold excess of biotin-dPEG(23)-NH_2_ (Iris Biotech) via microbial transglutaminase (Zedira) (0.1 units/μl) or incubated (100 μm TU1.20) for 16 h at 37 °C with a 100-fold excess of biotin-dPEG(23)-NH_2_ and KalbTG (0.5 μm) as a control. To achieve quantitative biotinylation, TU1.20 tagged at the C terminus of each heavy chain with YRYRQGGGS (100 μm) was conjugated 16 h at 37 °C with a 25-fold excess of biotin-dPEG(23)-NH_2_ via KalbTG (0.5 μm). To assess amino-donor specificity, the YRYRQGGGS-tagged IgG was conjugated with either a 5-fold excess of biotin-dPEG(23)-NH_2_ (Iris Biotech) or K-tag-biotin label (Z-RYESKG-PEG27-K(biotin)) via KalbTG (0.5 μm). All conjugates were purified via Superdex 200 to remove enzyme and unconjugated label.

### TSK GFC300 SA-FLUO analytics

IgG biotinylation was assessed by complex formation with SA-FLUO. SA-FLUO–IgG-biotin complexes were analyzed by analytical size-exclusion chromatography (TOSOH GFC300). In detail, equal volumes (50 μl) of biotinylated IgG (*c* = 0.6 mg/ml) and SA-FLUO (*c* = 0.3 mg/ml) were mixed and incubated for 5 min at room temperature. 20 μl of 100 mm biotin were added. 25 μl of each sample were injected on a TSK GFC300 SW 7.8 × 150-mm column, and extinction profiles were monitored at 280 and 494 nm. Successful biotinylation is monitored by the existence of SA-FLUO–IgG-Biotin complexes having extinction profiles at 280 and 494 nm.

### Elecsys TSH immunoassay

Site-specifically labeled IgG-biotin conjugates of TU1.20 were used at a concentration of 2.5 μg/ml in original TSH buffer replacing the original reagent in the R1 compartment of the Elecsys immunoassay rackpack (Roche Diagnostics). Cal1 and Cal2 from the TSH CalSet (Roche Diagnostics) were analyzed by the TSH Elecsys immunoassay (Roche Diagnostics) on a Cobas E170 module.

## Author contributions

W. S. and M. S. conceived and coordinated the study. W. S., F. C. K., J. P., V. L., T. J. A., and M. S. designed, performed, and analyzed the experiments shown in Figs. 1 and 2. W. S. designed, performed, and analyzed the experiments shown in Fig. 3. T. O. designed and analyzed the experiments shown in Fig. 4. J. B. and M. G. R. crystallized KalbTG enzyme; generated the PDB file; and designed, performed, and analyzed the experiments shown in Fig. 5. F. B. designed and synthesized the chemical labels and peptides used in all experiments and provided technical assistance. W. S., F. K., T. S., P. K., and M. B.-D. designed experiments and expressed, purified, and analyzed KalbTG enzyme. All authors reviewed the results, contributed to the writing of the paper, and approved the final version of the manuscript.

## Supplementary Material

Supplemental Data

## Footnotes

3 Please note that the JBC is not responsible for the long-term archiving and maintenance of this site or any other third party hosted site.

4 J. Patel, V. Lyamichev, and T. Albert, manuscript in preparation.

## Footnotes

2 The abbreviations used are:

MTG: microbial transglutaminase
KalbTG: MTG from *K. albida*
Z: benzyloxycarbonyl
Fmoc: *N*-(9-fluorenyl)methoxycarbonyl
SA-FLUO: fluorescein-labeled streptavidin
GLDH: glutamate dehydrogenase
Bi: biotin
PDB: Protein Data Bank.
