# Supplementary material for: Discovery of a microbial transglutaminase enabling highly site-specific labeling of proteins
Source: J Biol Chem. 2017 Jul 27;292(38):15622–35. doi: 10.1074/jbc.M117.797811 (PMC5612097; doi:10.1074/jbc.M117.797811)
Supplement: Supplemental Data [file supp_292_38_15622__index.html]

Discovery of a microbial transglutaminase enabling highly site-specific labeling of proteins — Discovery of a microbial transglutaminase enabling highly site-specific labeling of proteins — New transglutaminase for site-specific coupling — Supplemental Data 

# Discovery of a microbial transglutaminase enabling highly site-specific labeling of proteins

## Supplemental Data

- Supporting Information (.docx, 3.1 MB) - Supporting Information
