# Supplementary material for: Discovery of a microbial transglutaminase enabling highly site-specific labeling of proteins
Source: J Biol Chem. 2017 Jul 27;292(38):15622–35. doi: 10.1074/jbc.M117.797811 (PMC5612097; doi:10.1074/jbc.M117.797811)
Supplement: Supplemental Data [file 10.1074_M117.797811_jbc.M117.797811-1.docx]

**Supporting information**

**Supporting Table 1 KalbTG Glutamine and Alkyl-amine substrate preference on array and in solution assay**

| **Glutamine substrates** | | |
| --- | --- | --- |
| 5mer-Sequence | Array signal (arbitrary units) | Reaction rate in solution (pmol/s) |
| YRYRQ | 354 | 3.52 ± 0.08 |
| RYRQR | 344 | 3.60 ± 0.12 |
| RYSQR | 333 | 3.22 ± 0.10 |
| FRQRQ | 333 | 3.07 ± 0.17 |
| RQRQR | 304 | 2.06 ± 0.08 |
| FRQRG | 298 | 2.11 ± 0.13 |
| QRQRQ | 282 | 2.98 ± 0.01 |
| YKYRQ | 262 | 4.00 ± 0.18 |
| QYRQR | 262 | 1.92 ± 0.07 |
| DYALQ | Not detectable | Not detectable |
| MLAQG | NA | Not detectable |
| **Alkyl-amine substrates** | | |
| 5mer-Sequence | Array signal (arbitrary units) | Reaction rate in solution (pmol/s) |
| RYSKY | 5118 | 3.89 ± 0.04 |
| RYESK | 4656 | 4.47 ± 0.16 |
| AYRTK | 4310 | 3.65 ± 0.17 |
| RYRSK | 3824 | 3.26 ± 0.10 |
| RYGKS | 3559 | 2.66 ± 0.11 |
| YKGRG | 3100 | 3.01 ± 0.09 |
| Cadaverine | NA | 3.51 ± 0.12 |
| ARSKL | 2325 | 3.87 ± 0.31 |

**Glutamine substrate screening** KalbTG activity was obtained by measuring incorporation of N-(Biotinyl)cadaverine on the peptide array and rates of NADH oxidation at 340 nm and 37 °C in the presence of 500 µM cadaverine in the GLDH-coupled assay using 100 µM each of 9 of the best-performing array-selected Gln-substrates and of 2 MTG Gln-substrates. Strong correlation between the top array-selected substrates and their performance in the GLDH-coupled assay was observed, whereas KalbTG exhibited no activity with preferred MTG substrates DYALQ and MLAQG. Data is shown with background subtracted, single measurement representative of at least two replicates (array) or the average of triplicates with standard deviations (in-solution) are shown. Not detectable means readout was not significantly above background level. Array data for MLAQG is not applicable as Met-containing peptides were not synthesized on the array.

**Alkyl-amine substrate screening** KalbTG activity was obtained by measuring incorporation of Z-APRYRQRAAGGG-PEG-Biotin on the peptide array and rates of NADH oxidation at 340 nm and 37 °C in the presence of Z-GGGYRYRQGGGG (200 µM) in the GLDH-coupled assay using 100 µM each of 6 of the best-performing array-selected Lys-substrates, of cadaverine and of preferred MTG Lys-substrate ARSKL. Data is shown with background subtracted, single measurement representative of at least two replicates (array) or the average of triplicates with standard deviations (in-solution) are shown. Array data for cadaverine is not applicable as only peptides were screened on the array.

**Supporting Table 2 Data collection and refinement statistics.**

| Data statistics (PDB-ID 5M6Q) |  |
| --- | --- |
| Wavelength (Å) | 1.0 |
| Resolution range (Å) | 38.69 - 1.98 (2.05 - 1.98) |
| Space group | P 3 |
| Unit cell (Å, °) | A = 106.9 c = 56.1 |
| Total reflections | 244642 (23235) |
| Unique reflections | 49846 (4993) |
| Multiplicity | 4.9 (4.7) |
| Completeness (%) | 100 (100) |
| Mean I/σ (I) | 7.41 (1.10) |
| Wilson B-factor (Å^2^) | 29.1 |
| R-merge | 0.179 (1.48) |
| R-meas | 0.201 (1.68) |
| CC1/2 | 0.993 (0.327) |
| CC* | 0.998 (0.702) |
| <I^2^>/<I>^2^ | 2.0 |
| <\|E^2^-1\|> | 0.731 |
| Model refinement |  |
| Reflections used in refinement | 49846 (4984) |
| Reflections used for R-free | 2419 (318) |
| R-work | 0.183 (0.313) |
| R-free | 0.230 (0.350) |
| CC(work) | 0.967 (0.612) |
| CC(free) | 0.941 (0.575) |
| Number of non-hydrogen atoms | 4262 |
| macromolecules | 3765 |
| ligands | 75 |
| Protein residues | 450 |
| RMS(bonds) (Å) | 0.007 |
| RMS(angles) (°) | 1.08 |
| Ramachandran favoured (%) | 99 |
| Ramachandran allowed (%) | 1.3 |
| Ramachandran outliers (%) | 0 |
| Rotamer outliers (%) | 0.5 |
| Clashscore | 2.94 |
| Average B-factor (Å^2^) | 33.4 |
| macromolecules | 32.4 |
| ligands | 51.8 |
| solvent | 38.4 |

Statistics for the highest-resolution shell are shown in parentheses.

**
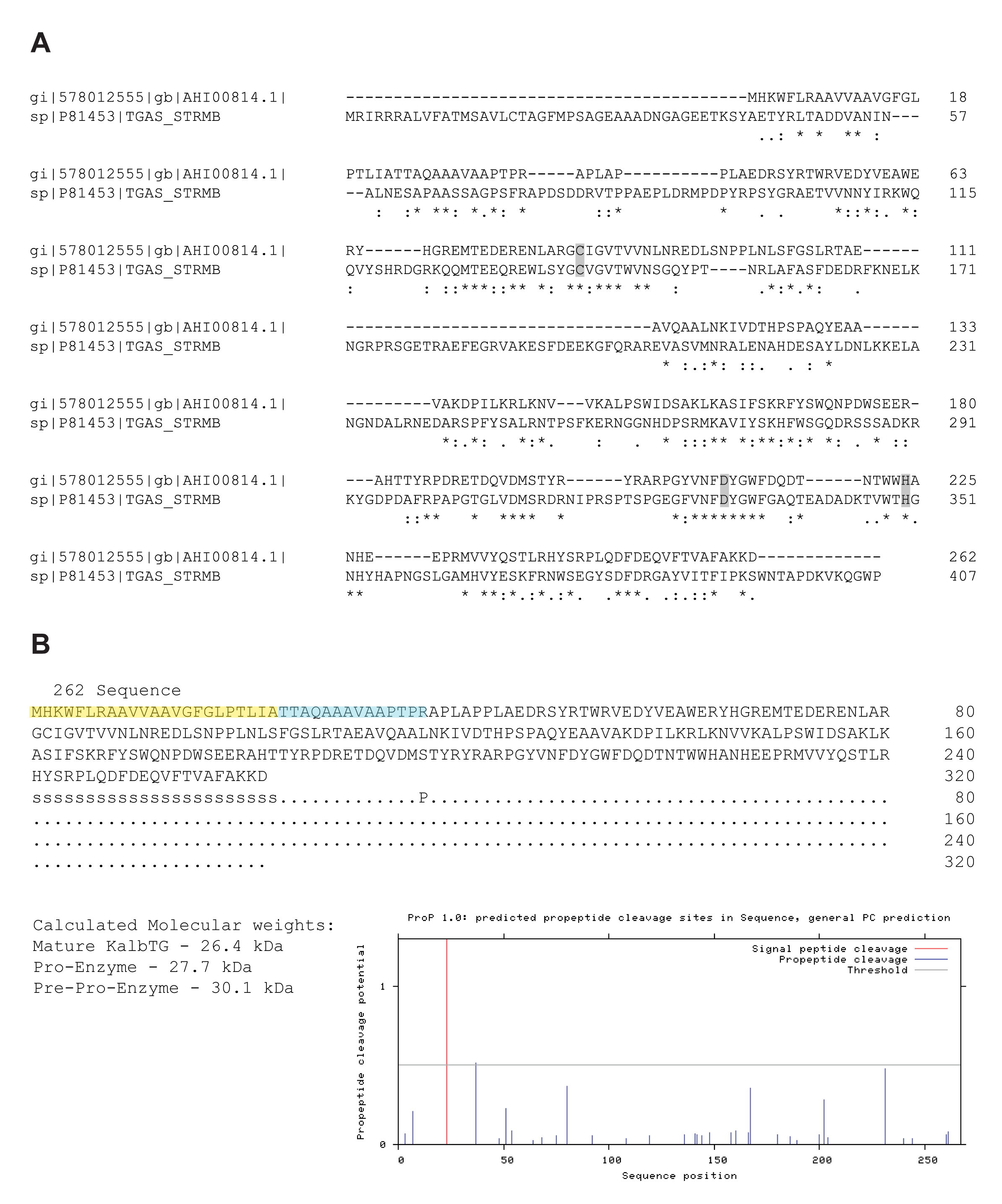
**

**Supporting Figure 1 Sequence analysis of *Kutzneria albida* microbial transglutaminase** A: Clustal O(1.2.1) multiple sequence alignment of *Kutzneria albida* hypothetical protein (GenBank AHI00814.1, upper row) and *Streptomyces mobaraensis* microbial transglutaminase (MTG, lower row). Identical amino acid residues are marked by asterisks, similar residues by colons. Conserved residues of the MTG catalytic triad (Cys, Asp, His) are highlighted. B: Amino acid sequence and calculated molecular weight of the hypothetical transglutaminase from *Kutzneria albida.* General cleavage site prediction (ProP 1.0), predicted signal peptide and propeptide sequences are indicated by a yellow or blue color respectively

**
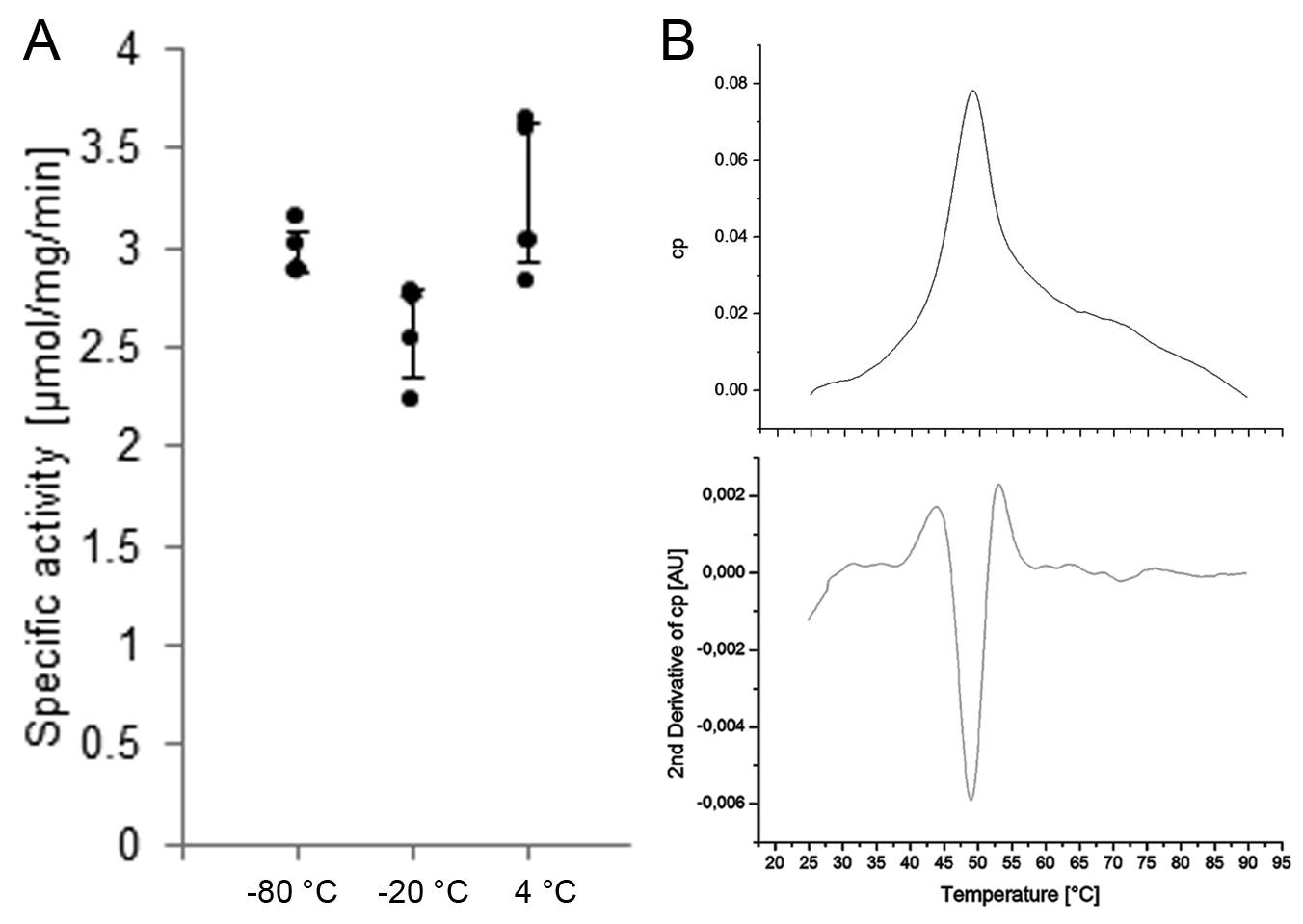
**

**Supporting Figure 2 Stability of KalbTG** A: Storage stability of the SlyD-fused enzyme functionally assessed by the GLDH-coupled assay. -80 °C; specific activity (2.98 ± 0.11 µmol/mg/min) of lab lot 2, stored at -90 to -60 °C and frozen/thawed once. -20 °C; specific activity (2.57 ± 0.22 µmol/mg/min) of lab lot 2, stored at -25 to -15 °C and frozen/thawed 5 times. 4 °C; specific activity (3.27 ± 0.35 µmol/mg/min) of lab lot 1, stored at 2 to 8 °C for 18 months. Lab lots 1 and 2 are from the identical purified protein preparation; lot 1 was immediately activated by dialysis, whereas lot 2 was first stored at -80 °C for 12 months in the presence of ammonium sulfate and then thawed and dialyzed. All measurements were performed in 4 replicates (4 wells on 96-well plate), shown are individual values with standard deviation as error bars. B: DSC analysis (heat capacity cp and second derivative of cp) of the mature enzyme. Tm was 48.9 °C.

**
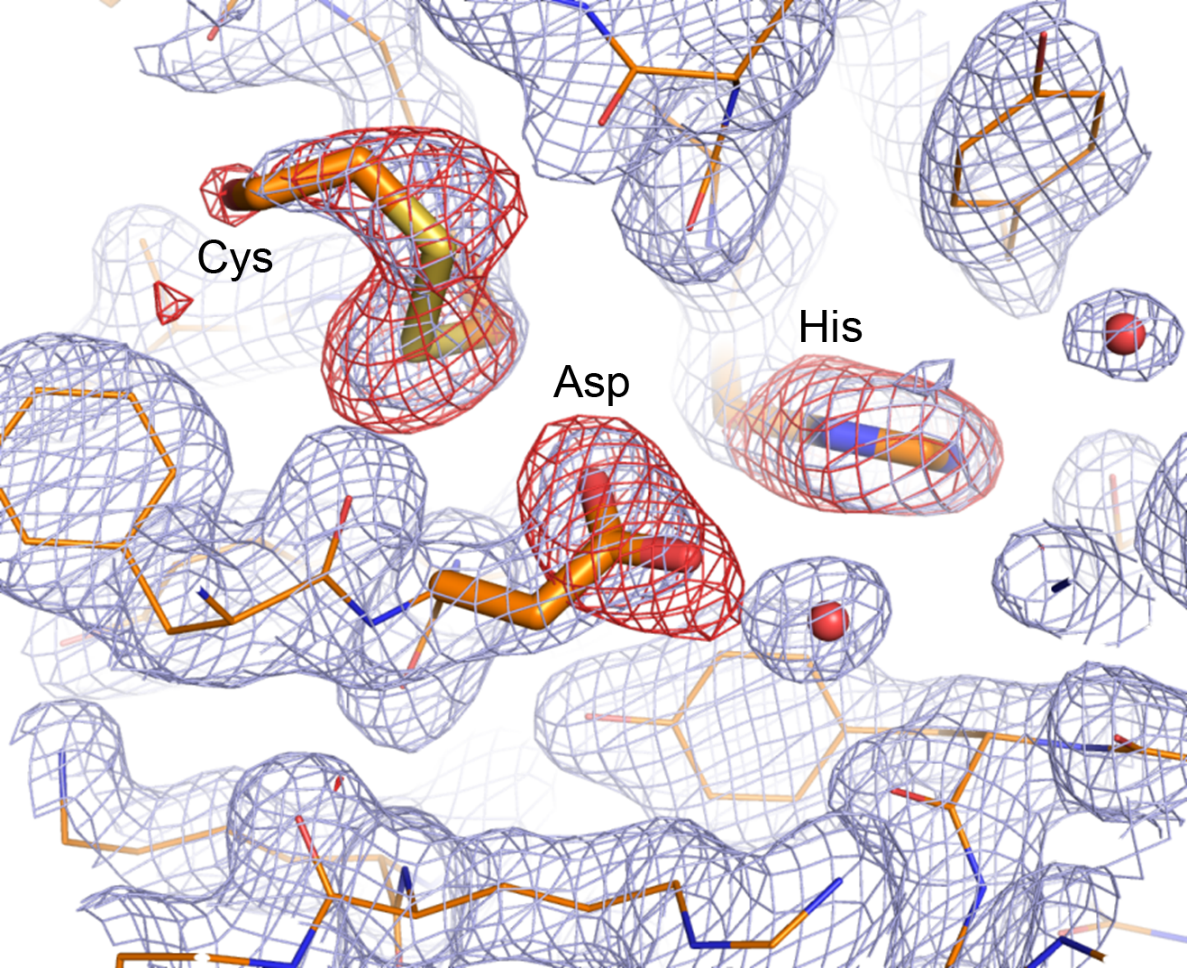
**

**Supporting Figure 3 Electron density** blue: 2Fo-Fc density at 1 rmsd 10 Å radius; red: Fo-Fc density at 3 rmsd of the catalytic triad, mutated to Ala. It is clear that the active Cys is partially modified, potentially by a cysteamine or β-mercaptoethanol

**
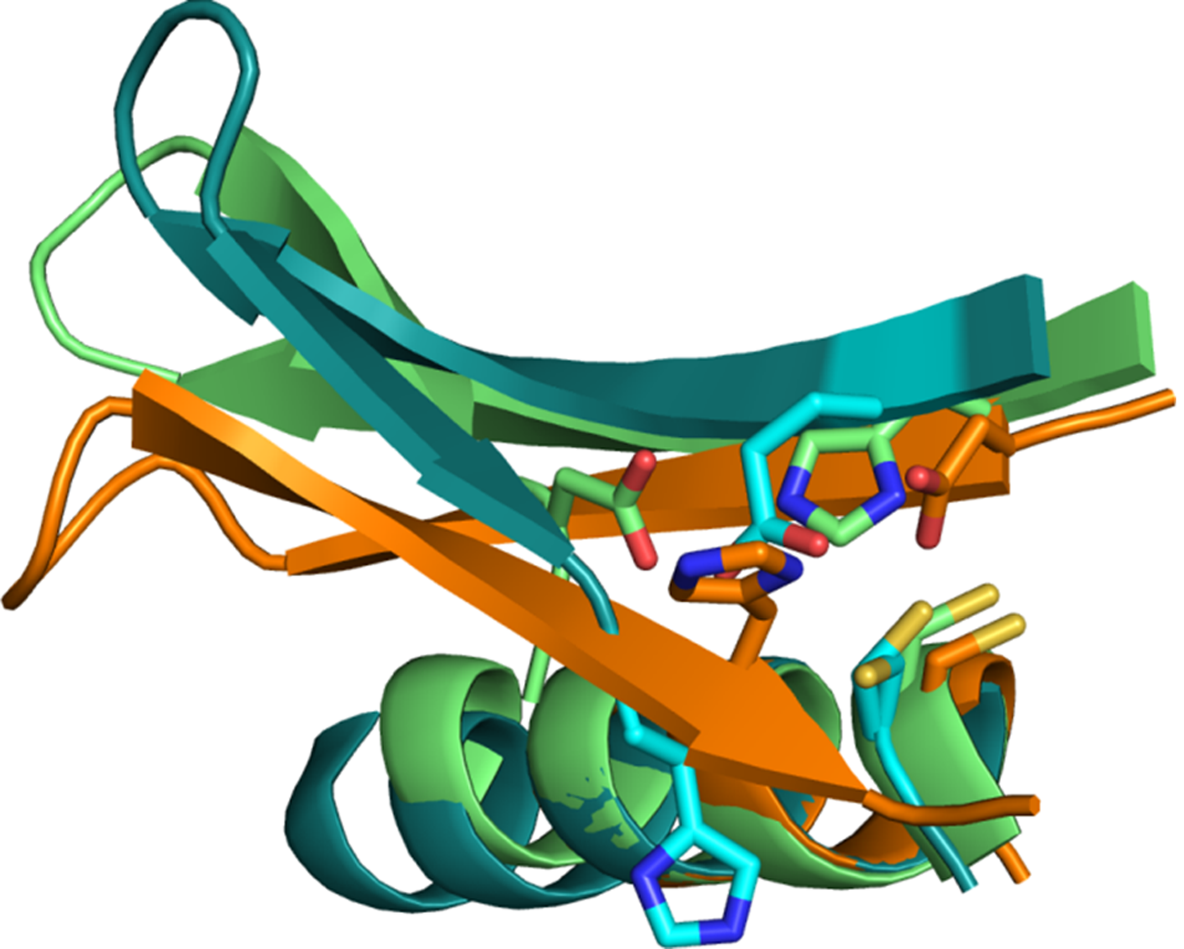
**

**Supporting Figure 4 Catalytic triads of other MTG** green: *S. suis* (PDB-ID 4xz7) – Cys-His-Asp; blue: *B. subtilis* (4p8i) – Cys-Glu-His; orange: KalbTG (5m6q, this work) – Cys-Asp-His. The three structures are very different, indicative of convergent evolution.
